# Programmatic Cost Evaluation of Nontargeted Opt-Out Rapid HIV Screening in the Emergency Department

**DOI:** 10.1371/journal.pone.0081565

**Published:** 2013-12-31

**Authors:** Jason S. Haukoos, Jonathan D. Campbell, Amy A. Conroy, Emily Hopkins, Meggan M. Bucossi, Comilla Sasson, Alia A. Al-Tayyib, Mark W. Thrun, For the Denver ED HIV Opt-Out Study Group

**Affiliations:** 1 Department of Emergency Medicine, Denver Health Medical Center, Denver, Colorado, United States of America; 2 Department of Emergency Medicine, University of Colorado School of Medicine, Aurora, Colorado, United States of America; 3 Department of Epidemiology, Colorado School of Public Health, Aurora, Colorado, United States of America; 4 Department of Clinical Pharmacy, Skaggs School of Pharmacy and Pharmaceutical Sciences, Aurora, Colorado, United States of America; 5 Department of Health and Behavioral Sciences, University of Colorado Denver, Denver, Colorado, United States of America; 6 Center for AIDS Prevention Studies, University of California San Francisco, San Francisco, California, United States of America; 7 Denver Public Health, Denver, Colorado, United States of America; Rollins School of Public Health, Emory University, United States of America

## Abstract

**Background:**

The Centers for Disease Control and Prevention recommends nontargeted opt-out HIV screening in healthcare settings. Cost effectiveness is critical when considering potential screening methods. Our goal was to compare programmatic costs of nontargeted opt-out rapid HIV screening with physician-directed diagnostic rapid HIV testing in an urban emergency department (ED) as part of the Denver ED HIV Opt-Out Trial.

**Methods:**

This was a prospective cohort study nested in a larger quasi-experiment. Over 16 months, nontargeted rapid HIV screening (intervention) and diagnostic rapid HIV testing (control) were alternated in 4-month time blocks. During the intervention phase, patients were offered HIV testing using an opt-out approach during registration; during the control phase, physicians used a diagnostic approach to offer HIV testing to patients. Each method was fully integrated into ED operations. Direct program costs were determined using the perspective of the ED. Time-motion methodology was used to estimate personnel activity costs. Costs per patient newly-diagnosed with HIV infection by intervention phase, and incremental cost effectiveness ratios were calculated.

**Results:**

During the intervention phase, 28,043 eligible patients were included, 6,933 (25%) completed testing, and 15 (0.2%, 95% CI: 0.1%–0.4%) were newly-diagnosed with HIV infection. During the control phase, 29,925 eligible patients were included, 243 (0.8%) completed testing, and 4 (1.7%, 95% CI: 0.4%–4.2%) were newly-diagnosed with HIV infection. Total annualized costs for nontargeted screening were $148,997, whereas total annualized costs for diagnostic HIV testing were $31,355. The average costs per HIV diagnosis were $9,932 and $7,839, respectively. Nontargeted HIV screening identified 11 more HIV infections at an incremental cost of $10,693 per additional infection.

**Conclusions:**

Compared to diagnostic testing, nontargeted HIV screening was more costly but identified more HIV infections. More effective and less costly testing strategies may be required to improve the identification of patients with undiagnosed HIV infection in the ED.

## Introduction

Over 1.1 million individuals are infected with HIV in the United States, while approximately 230,000 remain undiagnosed and 50,000 new infections occur annually [1], [2]. Testing for HIV infection is the first in a series of important interventions aimed at impacting the epidemic [3]. Identifying individuals with HIV infection provides a critical opportunity to link them into care where treatment slows the progression of disease and reduces infectivity [4], [5].

Recent policy recommendations have converged to include routine, non-risk-based HIV screening for most patients who seek medical care, including in emergency departments (EDs). In 2006 the Centers for Disease Control and Prevention (CDC) dramatically shifted its HIV testing paradigm to recommend nontargeted opt-out HIV screening in settings where the undiagnosed HIV prevalence was 0.1% or greater [6]. In 2013, the U.S. Preventive Services Task Force (USPSTF) published Grade A recommendations supporting routine HIV screening [7]. Both recommendations are congruent with the 2010 National HIV/AIDS Strategy and align with HIV testing proposed as part of the Patient Protection and Affordable Care Act [8].

Emergency departments are a major focus of HIV testing efforts in the United States, prompted by: (1) over 120 million ED visits occur annually [9]; (2) they serve substantial numbers of underserved patients [10]; and (3) they are the most common site of missed opportunities for diagnosing HIV infection [11]. However, most EDs in the United States still rely primarily on physician-directed diagnostic testing, with relatively sparse uptake of screening [12]. Slow translation has been attributed to a significant discordance between public health policy recommendations and the relative lack of empiric evidence to drive these recommendations [13], and the difficulty and costs of program implementation [14].

Although studies have demonstrated cost effectiveness of HIV screening from a societal perspective [15], [16], only a few have focused on direct programmatic costs of ED-based HIV screening [17], [18], [19], [20] and none have directly compared strategies as part of a clinical trial. To better inform operational considerations among emergency physicians and administrators, we proposed the following objectives as part of the Denver ED HIV Opt-Out Study: [21] (1) to estimate total direct costs associated with performing nontargeted opt-out rapid HIV screening in the ED per newly-identified HIV-infected patient; and (2) to compare such costs to those associated with diagnostic rapid HIV testing.

## Methods

### Ethics Statement

This study was approved by the Colorado Multiple Institutional Review Board with a full waiver of informed consent under 45 CFR 46.116(d). Therefore, patients did not provide written or verbal consent to participate in the screening portion of this study. However, patients provided verbal consent for HIV testing as part of standard medical care.

### Study Design

The design and results of the primary study have been reported elsewhere [21]. The current study was nested in a large prospective quasi-experiment to evaluate the clinical effectiveness of nontargeted opt-out rapid HIV screening in the ED when compared to diagnostic rapid HIV testing. Nontargeted screening (intervention) and diagnostic testing (control) were alternated in sequential four-month time intervals from April 15, 2007 through April 15, 2009 using a quasi-experimental equivalent time-samples design. Both HIV testing approaches were fully integrated into ED operations on a 24-hour basis using only existing ED and hospital staff.

### Setting

The current study was performed in the ED at Denver Health Medical Center in Denver, Colorado. Denver Health Medical Center is a 477-bed urban, public safety-net hospital with approximately 55,000 annual adult ED patient visits. It is also a regional level 1 trauma center and a nationally-recognized model for the integration of a public hospital, community health center clinics, and a public health department [22]. Large proportions of patients who present to Denver Health Medical Center are racial or ethnic minorities or socio-economically disadvantaged.

### Study Population

All patients ≥16 years of age and capable of providing consent for general emergency medical care were eligible to receive HIV testing. During the intervention phase, all eligible patients were offered rapid HIV testing using a non-risk-based approach and opt-out consent by registration personnel; during the control phase, patients identified as being at risk for HIV infection by their treating physicians were offered rapid HIV testing using an opt-in consent approach. Patients were excluded from HIV testing if they were: (1) unable to provide consent as determined by registration or clinical staff (e.g., altered mentation or requiring urgent or emergent evaluation or intervention); (2) prisoners or detainees; (3) victims of sexual assault; (4) sought care as a result of an occupational exposure; (5) self-identified as being infected with HIV; or (6) left the ED prior to being placed in a treatment room.

### Study Phases

The intervention phase consisted of three four-month periods during which nontargeted rapid opt-out HIV screening was performed 24-hours per day and using only existing ED and hospital staff. Registration staff obtained general medical consent from all patients and additionally offered voluntary and free rapid HIV testing to those who met criteria for inclusion using an opt-out consent approach. Consent for HIV testing was integrated into the general medical consent and required the patient to check a box and provide a signature indicating his or her decision to opt out. For patients who agreed to HIV testing, registration personnel triggered an automatic order using the electronic ED patient tracking system (Emergency Medical Services Information System [EMeSIS], Denver Health, Denver, CO). This system was available to all ED staff. Nurses and healthcare technicians used EMeSIS to identify patients who agreed to HIV testing and obtained a blood sample, which was sent it to the hospital's laboratory for rapid HIV testing. For patients who opted out during registration, physicians had the opportunity to diagnostically test them.

Rapid HIV testing was performed using a sequential algorithm to maximize the predictive accuracy of testing when performing a large number of screening tests. Whole blood was first tested using the Uni-Gold Recombigen HIV Test (Trinity Biotech, Wicklow, Ireland). Given the reported 100% sensitivity of this test, if it was negative, no other rapid test was performed, the result was reported as nonreactive, and the patient was considered HIV seronegative. If the first test was reactive, a second test was immediately performed using the OraQuick Advance Rapid HIV-1/2 Antibody Test (OraSure Technologies, Inc., Bethlehem, PA) in an effort to minimize false-positive results. If the second test was positive, patients were considered preliminarily positive for HIV infection. If the second test was negative, it was followed by a third rapid test, the Multispot HIV-1/HIV-2 Rapid Test (Bio-Rad Laboratories, Redmond, WA), to serve as a “tie breaker”. Because the diagnostic accuracy of this multiple rapid testing algorithm was also evaluated as part of this study, any reactive HIV test result was considered preliminary positive and all such patients were notified and referred into care. Confirmatory Western blot testing was performed once patients were linked into care.

The control phase consisted of three four-month periods during which physician-directed diagnostic rapid opt-in HIV testing was performed 24-hours per day using only existing ED and hospital staff [23]. Consent was obtained directly by physicians and documented in the patient's medical record. The physician then ordered a rapid HIV test using conventional methods of ordering diagnostic blood tests in the ED. Nurses or healthcare technicians obtained a blood sample and sent it to the laboratory for rapid HIV testing using the same sequential algorithm as in the intervention phase.

### Measurements

Direct programmatic costs were measured using actual costs and included: startup costs (e.g., computers, software, training, etc.); labor for administrative, ED, and laboratory staff; and supplies and equipment (e.g., HIV test kits, consent forms, informational sheets, blood draw supplies, etc.). Time-motion data collection was performed to determine labor costs associated with each of the two HIV testing methods [24]. A trained investigator performed all time-motion data collection using a structured, closed-response data collection instrument with a minimum of 30 observations made for each component of the testing process. Actual median hospital salaries were used to estimate labor costs, and all other direct costs, including startup costs, test kit costs, and other supplies were calculated using actual costs associated with program implementation. Finally, confirmed newly-diagnosed HIV infection was used as the outcome for this study.

### Data Management and Statistical Analyses

Patient and HIV testing data were electronically transferred from EMeSIS into an electronic database (Microsoft Access, Microsoft Corporation, Redmond, WA). Additionally, cost data were manually entered into an electronic spreadsheet (Microsoft Excel, Microsoft Corporation, Redmond, CA) and all analyses were performed using either Microsoft Excel or SAS Version 9.2 (SAS Institute, Inc., Cary, NC). Because of the equivalent time-samples design, observations from the three four-month opt-out periods and the three four-month diagnostic periods were combined into aggregate groups representing the nontargeted (intervention) and diagnostic (control) phases, respectively. Descriptive statistics were performed for all data. Medians and interquartile ranges (IQRs) were used to describe continuous data and percentages and 95% confidence intervals (95% CIs) were used to describe categorical data.

An economic evaluation from the ED perspective was performed to compare the two HIV testing methods. We specifically chose the hospital's perspective, rather than a societal perspective, to provide programmatic costs of both testing approaches for the benefit of healthcare policy makers and hospital administrators, knowing that it is important for them to understand the economic consequences related to each HIV screening approach if one is to be adopted as a part of routine care. Cost effectiveness ratios (CERs), or the total costs per patient identified with HIV infection, and the incremental cost effectiveness ratio (ICER), or the additional costs per patient identified with HIV infection above and beyond those incurred by diagnostic testing, were used to compare both testing programs.

We report the proportions and uncertainty (defined by the 95% CIs) of diagnosed HIV infection during each study phase, using the same methods as reported in our original article [21]. Given the relatively narrow precision of these estimates, we chose to hold the outcome fixed when performing the cost analyses. As such, we report uncertainty in the ICERs by conducting one-way sensitivity analyses using cost inputs and assumptions about these inputs. We report the most influential unit cost inputs and assumptions made to bias the findings away from diagnostic testing. We also performed a sensitivity analysis where HIV test kits were changed to $0 in order to simulate a scenario where HIV tests kits were fully reimbursed to the hospital by an external payer (i.e., the scenario generally supported by the Affordable Care Act). All costs were obtained and reported in 2009 dollars to correspond with the time period in which the study occurred [25].

## Results

**Figure 1:**
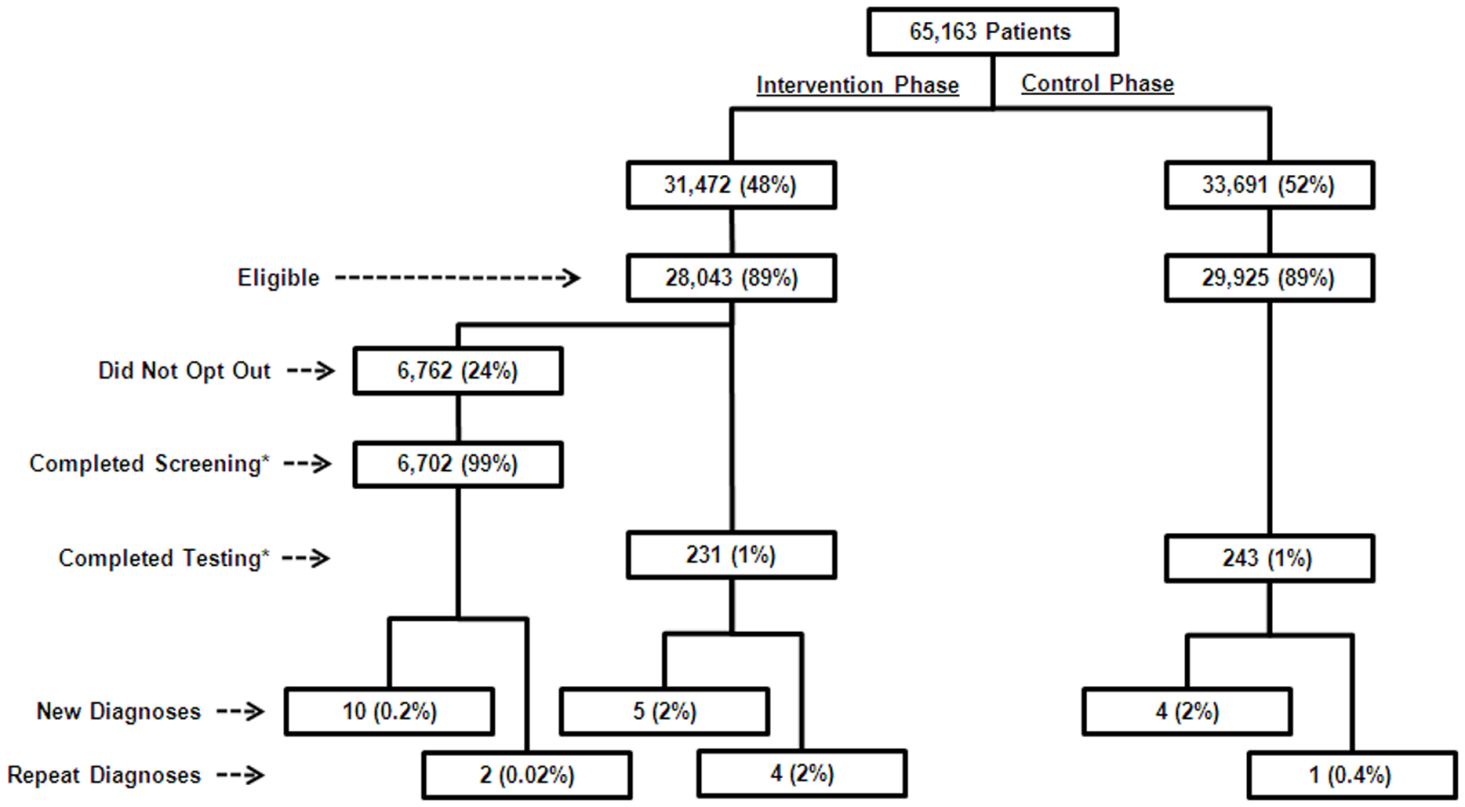
Patient flow diagram for the Denver Opt-Out Study. Eligible patients included those ≥16 years of age who were placed in an emergency department treatment room. (*Screening refers to HIV testing in conjunction with nontargeted opt-out screening, whereas testing refers to HIV testing in conjunction with physician-directed diagnostic testing.)

During the two-year study period, 65,163 patients presented to the ED, of which, 28,043 eligible patients were included in the intervention phase and 29,925 eligible patients were included in the control phase. Of those in the intervention phase, 6,762 (24%) did not opt-out and 6,702 (99%) were screened for HIV infection. Of the 6,702 patients, 10 (0.2%, 95% CI: 0.07%–0.3%) were newly-diagnosed with HIV infection. Of the 21,281 patients who opted-out, 231 (1%) were diagnostically tested by physicians, and 5 (2.2%, 95% CI: 0.7%–5.0%) were newly-diagnosed with HIV infection. Additionally, 723 repeat HIV tests were performed due to recidivism during the intervention phase, resulting in a total of 7,656 HIV tests performed. Of those in the control phase, 243 patients were diagnostically tested for HIV infection, and 4 (1.7%, 95% CI: 0.5%–4.2%) were newly-diagnosed with HIV infection. Additionally, 17 repeat HIV tests were performed due to recidivism during the control phase, resulting in a total of 260 HIV tests performed (**Figure 1**. Patient flow diagram for the Denver Opt-Out Study. Eligible patients included those ≥16 years of age who were placed in an emergency department treatment room. [*Screening refers to HIV testing in conjunction with nontargeted opt-out screening, whereas testing refers to HIV testing in conjunction with physician-directed diagnostic testing.]).

The annualized direct costs of nontargeted screening and diagnostic testing were $148,977 and $31,355, respectively (**Table 1**), and the costs per person tested during these phases were $19 and $121, respectively. During the intervention phase, 50% of all costs were incurred by purchasing rapid HIV tests and 37% by personnel time. In contrast, personnel time represented the highest proportion (32%) of incurred costs during the control phase. The difference in annualized direct costs of nontargeted screening and diagnostic testing was $117,622.

**Table 1:** Program costs of emergency department nontargeted opt-out rapid HIV screening and physician-directed diagnostic rapid HIV testing from the Denver ED HIV Opt-Out Study.

		Intervention Phase		Control Phase	
Cost Variable	Unit Cost (U.S. Dollars)	Number of Units	Cost (U.S. Dollars)	Number of Units	Cost (U.S. Dollars)
Startup					
Computer software	N/A	N/A	$1,884.00	N/A	$0.00
ED and laboratory staff training*	N/A	N/A	$7,687.31	N/A	$1,694.19
Personnel					
ED and laboratory staff time	N/A	N/A	$24,007.97	N/A	$10,672.54
Administrative staff time	N/A	N/A	$31,272.80	N/A	$15,636.40
Tests					
Uni-Gold rapid test	$9.50	7,656	$72,732.00	260	$2,470.00
Oraquick rapid test	$11.60	34	$394.40	5	$58.00
Multispot rapid test	$31.00	6	$186.00	0	$0.00
Confirmatory WB test	$110.00	34	$3,740.00	5	$550.00
Supplies and Equipment					
Blood draw supplies					
Entire blood draw kit	$1.05	2,761	$2,899.05	260	$273.00
Blood tube only	$0.06	4,895	$293.70	0	$0.00
Other supplies and printing					
Opt-out consent form	$0.08	47,309	$3,784.72	N/A	$0.00
Patient information sheet	$0.002	47,309	$94.62	N/A	$0.00
Diagnostic testing consent form	$0.002	231	$0.46	260	$0.52
**TOTAL**			**$148,977.03**		**$31,354.65**

Abbreviations: N/A = not applicable; ED = emergency department; WB = western blot.

Includes personnel time for trainers, trainees, and training supplies.

The CER of nontargeted screening for identifying patients with newly-diagnosed HIV infection was $9,932, whereas the CER of diagnostic testing was $7,839. Compared to diagnostic HIV testing, nontargeted HIV screening identified 11 additional newly-diagnosed HIV infections at a cost of $10,693 per additional new infection identified (ICER) (**Table 2**).

**Table 2:** Cost effectiveness ratios (CER) and the incremental cost effectiveness ratio (ICER) of nontargeted opt-out rapid HIV screening and physician-directed diagnostic rapid HIV testing for patients newly diagnosed with HIV infection from the Denver ED HIV Opt-Out Study, Denver, Colorado.

	Total Costs	Health Effect*	CER and ICER
Program	[C]	[E]	[C/E]
Non-targeted opt-out screening	$148,977.03	15	$9,931.80
Diagnostic testing	$31,354.65	4	$7,838.66
Incremental	ΔC	ΔE	ΔC/ΔE
	$117,622.38	11	$10,692.94

Defined as the number of patients newly-diagnosed with HIV infection.

The ICER comparing nontargeted HIV screening to diagnostic HIV testing was not sensitive to changes in unit cost inputs or other important cost assumptions. The most influential unit cost was the initial rapid HIV test cost. Varying the Uni-Gold Recombigen HIV Test unit cost by ±25% of the base-case ($9.50) changed the ICER to $9,096 and $12,290, respectively. Also, when the costs of HIV test kits were reduced to $0 for both study groups, the ICER became $3,968. Additional assumptions made to bias the findings away from diagnostic testing resulted in the following ICERs: (1) $9,977 assuming the same start-up costs between study groups; (2) $9,481 assuming the same ED and laboratory staff costs between study groups; and (3) $9,271 assuming the same administrative staff costs between study groups.

## Discussion

Nontargeted opt-out rapid HIV screening is more costly than physician-directed diagnostic rapid HIV testing in the ED, and when not taking into account potential uncertainty in effectiveness of the two approaches, diagnostic testing is more economically efficient per newly-diagnosed patient. This finding results from nontargeted screening incurring an approximate additional $118,000 in annualized direct costs while identifying a relatively small number of additional patients with HIV infection. Additionally, because of the relatively small difference in numbers of patients newly identified with HIV infection between the two study arms, the cost effectiveness of nontargeted screening was relatively insensitive to changes in costs.

This is the first study to compare direct programmatic cost effectiveness of nontargeted opt-out rapid HIV screening, the approach currently recommended by the CDC, to diagnostic HIV testing, the approach supported as a minimum standard of care in the ED [6], [26]. Notably, there have been studies that have evaluated cost effectiveness of HIV screening from a societal perspective, concluding that HIV screening is cost effective even in low prevalence populations [14], [15], [16]. Although these studies are important, extrapolation of these findings to EDs, where screening strategies and their effectiveness vary widely (and may depend on underlying prevalence of HIV infection in the base population), and where resources and preventive care priorities are limited, is difficult. To date, a relatively small number of studies have focused on programmatic costs of ED-based HIV screening [17], [18], [20], [27], and a smaller number have attempted to quantify the cost effectiveness of ED-based HIV screening [19], [20], [28]. Thus far, all ED-based studies have concluded that performing HIV screening or testing is cost effective, although comparative cost effectiveness studies have been extremely limited. When comparing costs per newly-identified HIV infection, reported estimates range from $1,780 to $9,688, both adjusted to 2009 dollars using the Consumer Price Index to correspond with our results [18], [27]. Moreover, to our knowledge, the only study to include programmatic costs using an “opt-out approach” reported $5,208 per newly-diagnosed infection, also adjusted to 2009 dollars [17]. Unfortunately, this latter study was limited by inclusion of only test kit costs and hourly wages of external HIV testing staff. Other studies, although useful in understanding costs associated with ED-based HIV screening, have also been limited by model assumptions (i.e., theoretical aspects of how well certain HIV screening components function in actual practice) [20] or have reported results as costs per quality-adjusted life-years [19], [20], [28], making it difficult to compare results of studies that have used a different outcome.

Although most studies have concluded that HIV screening is cost effective, cost effectiveness does not necessarily imply affordability. If additional resources are willing to be allocated to HIV screening (i.e., willingness-to-pay is at or above $10,693 per additional case detected), nontargeted screening becomes the preferred approach over diagnostic testing, operating under the assumption that more detection is preferred. This is particularly true when considering the importance of long-term costs and benefits of patients with known HIV infection who are maintained in care. During the nontargeted phase of our study, most of the costs were incurred by purchase of the rapid HIV test kits. It is conceivable as we move from a model in which free HIV testing is provided (i.e., HIV testing funded by the hospital) to one where reimbursement (via private insurance or other subsidy) occurs, this portion of programmatic costs may be reduced from the hospital perspective. However, under an insurance reimbursement model where hospitals would not have to pay for HIV test kits, the hospital-perspective ICER was reduced to $3,968 with nontargeted screening dominating diagnostic testing; unfortunately, in this sensitivity analysis the hospital perspective does not appropriately account for relevant costs absorbed by other payers.

With the recent synergy of HIV screening policies in the United States, including most recently the USPSTF Grade A recommendation for performing “routine HIV screening” [7], it will be imperative for ED and hospital administrators to critically evaluate and implement affordable HIV testing processes that are effective, scalable, and affordable. A critical next step includes understanding willingness-to-pay thresholds per unit outcome from payers' perspectives; until these thresholds are known, it will be impossible to define which HIV screening approach is most efficient. The National HIV/AIDS Strategy calls for an approximate 10% absolute reduction in the proportion of individuals with undiagnosed HIV infection by 2015 [29]. As EDs remain this country's primary medical care safety net, reaching this target will likely require broader ED-based HIV testing efforts than currently exist.

It still remains unclear which HIV screening approach or approaches are best suited for EDs and other clinical settings that are not principally developed for this form of preventive care. HIV screening approaches for this clinical setting requires identifying alternative methods that optimize the identification of newly-diagnosed HIV cases while balancing the use of scarce resources. Novel, empirically-developed, risk-based HIV screening has been recently shown to identify comparable numbers of patients with newly-diagnosed HIV infection while testing a significantly fewer number of patients as compared to nontargeted HIV screening [30]. The cost effectiveness of this approach, however, remains unknown.

## Limitations

This study used newly-diagnosed HIV infection as an intermediate outcome and therefore did not model costs relative to quality-adjusted life-years or other future health outcomes. Differences in the lifetime medical costs and transmissions averted between patients identified with HIV infection in the two study arms may impact cost effectiveness. Cost assumptions and inputs were not sensitive to the ICER (i.e., all ICERs derived from sensitivity analyses were greater than $7,839, the CER of diagnostic testing). Also, given the small number of outcomes, we did not use statistical methods (e.g., bootstrapping) to provide estimates of uncertainty for reported ICERs. We do believe, however, that reporting cost and effectiveness results from an actual clinical trial is important and contributes meaningfully to the broader knowledge base of HIV screening performance in EDs. Finally, costs analyses may be influenced by the HIV screening program, which was performed at a single institution and therefore may not be generalizable.

## Conclusions

Compared to diagnostic testing, nontargeted opt-out rapid HIV screening was more costly but identified more HIV infections. More effective and less costly testing strategies may be required to improve the identification of patients with undiagnosed HIV infection in the ED. Incorporation of lifetime medical costs and transmissions averted are required to assess the broader cost-effectiveness of the two testing methods.
